# Vitamin D and Cardiovascular Risk: Which Implications in Children?

**DOI:** 10.3390/ijms21103536

**Published:** 2020-05-16

**Authors:** Silvia Savastio, Erica Pozzi, Francesco Tagliaferri, Roberta Degrandi, Roberta Cinquatti, Ivana Rabbone, Gianni Bona

**Affiliations:** 1 SCDU of Pediatrics, Azienda Ospedaliero-Universitaria Maggiore della Carità, University of Piemonte Orientale, 28100 Novara, Italy; ericapozzi@yahoo.it (E.P.); cecco.taglia@gmail.com (F.T.); roberta.degrandi90@gmail.com (R.D.); roberta.cinquatti@gmail.com (R.C.); ivana.rabbone@uniupo.it (I.R.); gianni.bona@maggioreosp.novara.it (G.B.); 2 Department of Health Sciences, University of Piemonte Orientale, 28100 Novara, Italy

**Keywords:** childhood, vitamin D, cardiovascular risk, extra-skeletal effects

## Abstract

Vitamin D (25OHD) pleiotropic effects are widely recognized and studied. Recently, vitamin D cardiovascular effects are gaining interest, especially in children, although the studies present conflicting data. Some randomized controlled trials (RCTs) have demonstrated that cardiovascular risk markers, such as lipid parameters, inflammation markers, blood pressure, and arterial stiffness, are unaffected by vitamin D supplementation. By contrast, other studies show that low vitamin D levels are associated with higher risk of cardiovascular disease (CVD) and mortality, and support that increased risk of these diseases occurs primarily in people with vitamin D deficiency. An update on these points in pediatric patients is certainly of interest to focus on possible benefits of its supplementation.

## 1. Introduction

Epidemiological studies have found a significant inverse association between serum vitamin D (25OHD) levels and cardiac injury or hypertension. In vitro, vitamin D appears to suppress the intracellular NF-κB pathway and renin synthesis attenuating the progression of coronary artery disease [1]. This could be related to an inflammation increase due to vitamin D deficiency [2]. However, vitamin D supplementation did not clearly show cardiovascular improvements in adults and children/adolescents.

This review provides a summary of the actual knowledge of the role of vitamin D in cardiovascular disease (CVD) according to its pathophysiological aspects. Moreover, we discuss whether vitamin D supplementation may influence cardiovascular risk markers with its anti-inflammatory function. We included in this review only English language studies meeting these criteria: participants were children or adolescent; measured endpoints were blood pressure, lipid profile, pulse wave velocity, and CVD events. Only papers published from 1 January 2015 and 31 March 2020 were considered.

## 2. Vitamin D and the Cardiovascular Tissue: Pathophysiological Effects

Vitamin D is a group of fat-soluble hormones [3,4]. Two main forms exist in nature: ergosterol (provitamin D_2_) and 7-dehydrocholesterol (provitamin D_3_). The first is a steroid found primarily in fungi and plants, the latter is of animal origin and is produced in the skin by ultraviolet (UV) B radiation. Hence, vitamins D_2_ and D_3_ are available to human body coming from different sources: ambient UV exposure (vitamin D_3_), dietary intakes of vitamin D_3_-rich foods (egg yolks and oily fish), fortified foods (margarine and breakfast cereals, generally vitamin D_2_ fortification) and vitamin supplements [5]. Ultraviolet B radiation activates both provitamins to ergocalciferol and cholecalciferol, respectively [5]. Season, latitude, melanin, and sunscreen are factors strictly connected with the production. 

Vitamin D is transported to the liver by vitamin D binding protein (VDBP), where it is hydroxylated to 25-hydroxyvitamin D (25OHD), the major circulating form and the most reliable biomarker of the vitaminic status. Afterwards, VDBP transports 25OHD to the kidneys, where it is filtered by the glomerulus and uptaken in the tubular cells. In the kidney the enzyme 1-alpha hydroxylase (Cytochrome P450 27B1, CYP27B1) transforms again 25OHD into its active form, 1,25-dihydroxyvitamin [calcitriol, 1,25(OH)_2_D]. This form of vitamin D is also produced in other tissues due to the expression of CYP27B1: bowel cells, vascular smooth muscle cells, B lymphocytes, monocytes, dendritic cells, and other ones [6]; in these sites calcitriol seems to have paracrine-autocrine effect to regulate cell growth and differentiation [7]. From kidney, 1,25(OH)_2_D reaches cells in target organs through bloodstream bound to VDBP, then it passes through cellular membranes and, after binding cytosolic receptor (vitamin D receptor, VDR), it enters the nucleus and activates gene expression. Vitamin D receptor is a transcription factor regulating the expression of genes. It is a member of a large family of nuclear hormone receptors and it is not restricted to those tissues considered the classic target of vitamin D. The VDR-1,25(OH)_2_D complex heterodimerizes with other nuclear hormone receptors and binds to special DNA sequences called vitamin D response elements (VDREs) [4]. Thousands of VDREs in hundreds of genes have been described so far [8]. Therefore, this could explain the potential responsibility of vitamin D in the development of diabetes, cancers, autoimmune disorders, kidney disease, and other neurodegenerative disease [3]. 

From the mid-1980s it became clear that some of the actions of vitamin D were too rapid to be accounted for changes at the genomic level [9]. In the last decades, studies recognised that 1,25(OH)_2_D also exerts non-genomic actions, involving the activation of signaling molecules, such as phospholipase C and phospholipase A2, phosphatidylinositol-3 kinase and p21ras, and the rapid generation of second messengers (Ca^2+^, cyclic AMP, fatty acids, and phosphatidylinositol-3,4,5-trisphosphate), accompanied by the activation of protein kinases [9]. The non-genomic actions also include the opening of Ca^2+^ and Cl^-^ channels [10]. 

Vitamin D exerts many effects on calcium-phosphorus metabolism and has more extra-skeletal function, although some molecular mechanisms remain still unclear [11]. In recent years, it has become increasingly evident that 1,25(OH)_2_D regulates multiple cellular processes with effects on cell growth and differentiation, on the innate and adaptive immune function, and, getting to the point, on cardiovascular functionality [12].

**Figure 1:**
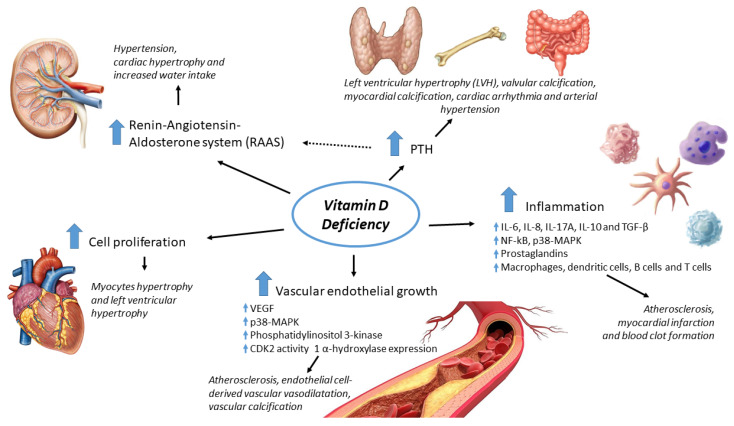
The main mechanisms involved in cardiovascular risk due to vitamin D deficiency.

The mechanisms by which vitamin D exerts its cardio and vasculoprotective effects are not fully understood yet. We stress the main pathways involved below (Figure 1). 

*Modulation of inflammation*—Vitamin D is known to be a powerful modulator of inflammation through different mechanisms. As mentioned above, vitamin D has been reported to inhibit NF-κB activity [13,14,15]: this inhibition has been shown to attenuate the development of cardiovascular complications and to induce cardio-protective effect [16,17]. Derakhshanian et al. [18] have reported that vitamin D could significantly decrease NF-kB activity in cardiomyocytes of diabetic rats, showing the potential key role of the vitamin in the cardiovascular health of people with diabetes. In addition, Al-Rasheed et al. [19] have demonstrated that the administration of cholecalciferol markedly attenuated the development of induced cardiac hypertrophy in mice probably through these signaling pathways.

Furthermore, vitamin D regulates the levels of cytokines including interleukins (IL-6, IL-8, IL-17A, IL-10) and TGF-β [20]. In addition, it inhibits the prostaglandins pathway via reducing their receptors, decreasing COX-2 expression and increasing 15-PGDH expression. Lastly, vitamin D inhibits the immune cells via VDR including macrophages, dendritic cells, B cells, and T cells [20]. These effects of 25OHD can contribute to the inhibition of various inflammatory mediated processes such as atherosclerosis, myocardial infarction and blood clot formation [21]. Vitamin D deficiency may also accelerate atherosclerosis through activation of endoplasmic reticulum stress of macrophages within the atherosclerotic plaque [22].

*Regulation of renin-angiotensin-aldosterone system (RAAS)*—RAAS is an important contributor to changes in arterial and cardiac stiffness, leading to hypertension and clinical heart failure [23]. In animal experiments, vitamin D was found to be a potent endocrine suppressor of renin biosynthesis: VDR -/- mice had elevated production of renin and angiotensin II, causing hypertension, cardiac hypertrophy, and increased water intake [24,25]. Chandel et al. [26] analyzed how VDR modulates the RAAS activity finding that vitamin D receptor deficit induces its activation through SIRT1/PPAR-c/VDR signaling in podocytes. Zittermann et al. [27] evaluated the effect of three years of vitamin D supplementation (4000 IU daily) on parameters of the RAAS (renin and aldosterone) in 165 patients with advanced heart failure, with a not significant change in RAAS parameters; nevertheless, the study showed an increase in serum renin concentrations in the subgroup with low baseline 25OHD levels. 

*Regulation of parathormone (PTH)*—Vitamin D inhibits production of PTH through a feedback mechanism. Chronic vitamin D deficiency reduces intestinal calcium absorption and bone calcium mobilization leading to overproduction of parathyroid hormone. PTH may cause left ventricular hypertrophy (LVH), valvular calcification, myocardial calcification, cardiac arrhythmia, and arterial hypertension [28,29,30,31]. Some of these effects involve the activation of renin-angiotensin-aldosterone system [32].

*Regulation of cardiac myocyte proliferation and hypertrophy*—Vitamin D induces hypertrophy in immature and mature cardiac myocytes and inhibits proliferation blocking entry into the S phase of the cell cycle [33]. Lower 25OHD levels are associated with left ventricle hypertrophy [34,35].

*Regulation of vascular smooth muscle*—in vitro, studies support that 25OHD regulates endothelial cells proliferation and hypertrophy via different pathways: the release of vascular endothelial growth factor (VEGF) [36,37]; the modulation of tissue factor and protease-activated receptor 2 expression [38]; the activation of phosphatidylinositol 3-kinase [39]; the suppression of lipopolysaccharide-induced inflammatory response in vascular smooth muscle cells (VSMCs) via inhibition of the p38 MAPK signaling pathway [40]; the inhibition of VSMC proliferation through a Cdc25A-dependent (cell division cycle 25 homolog A) mechanism [41]. Moreover, vitamin D enhances endothelial cell-derived vascular vasodilatation [42]. Lastly, Torremadé et al. [43] have shown that vascular calcification in chronic kidney disease is mediated by an increase of 1alpha-hydroxylase expression in vascular smooth muscle cells.

## 3. Vitamin D Deficiency and Cardiovascular Risk Factors

Over the years, several observational studies in adults have found an association between low vitamin D levels and higher blood pressure levels, myocardial infarction, heart failure, coronary heart disease, peripheral arterial disease, and atherosclerosis [21,44,45].

Cardiovascular diseases represent a major cause of death and disability worldwide and affect a large portion of adults past the age of 60 years. Although overt disease in youth is rare, atherosclerotic process can begin early in childhood [46]. 

Clinical events such as myocardial infarction, stroke, peripheral arterial disease, and ruptured aortic aneurysm are the culmination of the lifelong vascular process of atherosclerosis. Pathologically, the process begins with the accumulation of abnormal lipids in the vascular intima, a reversible stage. It progresses to an advanced stage in which a core of extracellular lipid is covered by a fibromuscular cap, and culminates in thrombosis, vascular rupture, or acute ischemic syndromes.

In most of the pediatric population, atherosclerotic vascular changes are mild and can be minimized or even prevented through a healthy lifestyle. However, in some children, the process is accelerated due to the presence of identifiable cardiovascular risk factors such as dyslipidemia, hypertension, hyperglycemia, and obesity. All these conditions are considered as part of the metabolic syndrome [47], a condition of insulin resistance that predisposes to the development of cardiovascular diseases and type 2 diabetes mellitus. There are several definitions for metabolic syndrome; however, the clinical implication of such diagnosis is the identification of patients who need lifestyle interventions focused on increased physical activity and weight reduction. 

The prevalence of overweight and obesity in childhood and adolescence is steadily rising worldwide. Approximately one-third of children and adolescents in the United States are either overweight or obese [48]. This condition is due to both poor diet and a sedentary lifestyle [47].

Many recent studies have investigated the association between vitamin D deficiency and cardiovascular risk factors in overweight and obese children [49,50,51,52,53]. Some studies found a higher prevalence of dyslipidemia in vitamin D deficiency obese subjects compared to subjects with vitamin D sufficiency [50,51,52]. For example, Censani et al. [50] found that overweight and obese children with 25OHD deficiency (<20 ng/mL) had significantly higher non-High Density Lipoprotein (HDL) cholesterol (*p* < 0.03), total cholesterol (TC; *p* < 0.01), triglycerides (TG; *p* < 0.03), Low Density Lipoprotein (LDL) levels (*p* < 0.03), TG/HDL ratio (*p* = 0.03), and TC/HDL ratio (*p* < 0.01) than children with 25OHD ≥20 ng/mL. Iqbal et al. [51] examined a population of 376 children with severe obesity: c-HDL resulted lower in children with 25OHD < 30 ng/mL compared to those with 25OHD ≥30 ng/mL (*p* < 0.0001). No other correlations between TC and non-HDL cholesterol and 25OHD levels were found. Lee et al. [52] showed that lipid levels (total cholesterol, non-HDL cholesterol) and oxidized LDL levels were significantly inversely associated with 25OHD concentration in a population of 209 obese American children.

In contrast to before mentioned studies, Colak et al. [49] reported no relationship between vitamin D deficiency and dyslipidemia or abnormal glucose homeostasis. On the other hand, they showed that serum 25OHD levels were negatively associated with 24-h ambulatory blood pressure and carotid intima-media thickness (*p* < 0.05). Similarly, Kao et al. [53] detected that lower serum 25OHD levels were associated with higher systolic (*p* = 0.03) and diastolic (*p* = 0.009) blood pressures, even after adjustment for BMI. 

We must consider that obesity itself is associated to elevated cardiovascular risk factors and metabolic syndrome [47], and that 25OHD deficiency has a very high prevalence in obese subjects [49,50,51,52]. Indeed, obese individuals are sedentary, little exposed to sunlight and with poor diets, leading to lower vitamin D levels. In addition, vitamin D seems to be stored in adipose tissue [54].

In order to exclude the confounding factor due to body weight, Petersen et al. [55] considered the impact of fat mass and physical activity on the association between 25OHD levels and cardiometabolic markers. They found that each 10 mmol/L 25OHD increase was associated with lower diastolic blood pressure (*p* = 0.02), TC, c-LDL, TG (*p* ≤ 0.001 for all lipids), and lower metabolic syndrome score (*p* = 0.01). They observed that adjustment for fat mass index did not change the associations. Kim et al. [56] also studied a population of non-obese children and found higher TG levels and TG/c-HDL ratio in the vitamin D-deficient group (<20 ng/mL) than in the normal group (*p* = 0.03). Moreover, the vitamin D level was significantly inversely associated with TG level and TG/c-HDL (*p* < 0.001). Liang et al. [57] found a serum 25OHD level significantly lower in hypertensive subjects compared to controls (*p* = 0.02). In addition, they evaluated the level of 25OHD receptor that turned out to be lower in hypertensive children (*p* = 0.003). 

This seems to support the hypothesis that vitamin D level may affect the lipid profile, regardless of fat mass. 

Conversely, Baker et al. [58], evaluating a population of lean and active young adults aged 18–24 years from rural India, did not find a clear association between serum vitamin D levels and CVD risk factors, including blood pressures, arterial stiffness, carotid intima-media thickness fasting lipids, glucose, and insulin. They concluded that a vitamin D insufficiency may be considered a marker of unhealthy lifestyle (such as physical inactivity and obesity) rather than being causally related to cardiovascular disease risk.

Interestingly, some studies hypothesize that 25OHD may play a role in prenatal life in modifying CV risk through mechanisms still unknown [59,60]. 

For example, Arman et al. [59] focused on a population of 135 term healthy infants. They analyzed vitamin D values at birth and performed ultrasound measurements at 24–48 h after birth. Significant lower mean and maximum aortic intima-media thickness (IMT) measurements were found in children with 25OHD sufficiency. IMT value is a good predictor of increased risk of cardiovascular disease and atherosclerosis [61,62] 

Sauder et al. [60] measured total and bioavailable 25OHD in cord blood and in blood from 4- to 6-year-old children. They then assessed cardiovascular risk factors (blood pressure, arterial stiffness, body size, and adiposity) at 4 to 6 years. They observed significant inverse associations of 25OHD cord blood levels with childhood systolic (*p* < 0.01) and diastolic (*p* = 0.01) blood pressure. 

Wang et al. [63] analyzed the association between systolic blood pression (SBP) and 25OHD levels (measured both in cord blood and in early childhood) in a prospective birth cohort study of 775 children. Low vitamin D status at birth and a vitamin D insufficiency in early childhood were associated with elevated SBP at ages 3 to 18 years. 

Therefore, low vitamin D status appears a risk factor for hypertension also in pediatric age. It is possible that a vitamin D insufficiency in early life may modify fetal development and influence arterial structure and metabolic processes. 

However, Miliku et al. [64] did not observe an association between vitamin D and childhood cardiovascular risk factors evaluating a cohort of 4903 mothers and their offspring.

Table 1 shows the main significant studies on vitamin D deficiency and cardiovascular risk factors in pediatric age.

The biological mechanisms underlying these results are still not clear. We can suppose that, as stated above, a vitamin D deficiency increase inflammation in the body activating different pathways, which might lead to cardiac hypertrophy and increased CVD risk. To support this, in a condition as Kawasaki syndrome, the most common cause of acquired heart disease in children, low 25OHD serum concentrations were found in the subgroup who developed coronary artery abnormalities, suggesting how vitamin D might have a contributive role in the development of coronary artery complications [65]. 

## 4. The Impact of Vitamin D Supplementation 

The protective role of vitamin D for atherosclerosis and coronary arterial disease (CAD) has been documented in swine and mice models. The beneficial role of its supplementation after myocardial infarction has been showed in vitro studies [66,67,68]. However, most randomized controlled trials (RCTs) conducted in the adult population have shown no beneficial effects of vitamin D supplementation in preventing cardiovascular diseases or reducing cardiovascular risk [21,69,70,71,72,73]. It is still not clear whether calcitriol or other potent vitamin D analogues might have major effects and whether specific subgroups of patients, such as type 2 diabetic subjects with CAD, might benefit most from vitamin D supplementation [69,70,71,72,73].

As discussed previously, there is evidence of association between low levels of vitamin D and cardiovascular risk factors in children, which may lead to cardiovascular diseases in adulthood. Therefore, in recent years new randomized controlled studies examining effects of vitamin D supplementation on cardiovascular outcomes in children and adolescents have been performed [74,75,76,77,78,79,80,81,82,83,84].

In the last five years, six RCTs focused on obese nondiabetic adolescents (11–17 years) [79,80,81,82,83,84]. Shah et al. [80] failed both to increase 25OHD levels and to alter markers of inflammation and cardiovascular risk in a group of obese adolescents through a vitamin D supplementation of 150,000 IU every 3 months. 

Varshney et al. [84] performed a RCT in a large sample of patients aged 11–17 years, with long duration of intervention (12 months) and with a high dose of vitamin D supplementation (120,000 IU/month) achieving 25OHD levels > 20 ng/mL in 68% of subjects and > 30 ng/mL in 41.2% of subjects of the intervention group. Nevertheless, they could not find statistically significant differences neither in beta cell function, nor in cardiometabolic markers nor in lipid profile. Alike, the other five studies managed to significantly increase serum 25OHD levels after treatment without reaching cardiovascular endpoints [82,83,84].

However, two studies revealed improvement trends following vitamin D treatment [79,83] on CV markers. Sethuruman et al. [83] found a positive correlation between increase of 25OHD levels and increase of c-HDL after vitamin D supplementation in obese adolescents with baseline 25OHD level < 20 ng/mL. In addition, ergocalciferol supplementation seemed to have a beneficial impact on fasting insulin without changes in Homeostasis Model Assessment of Insulin Resistance (HOMA-IR). Brar et al. [79] observed that using a high dose of ergocalciferol in a cross over design study increased whole body insulin sensitivity in the treated group (*p* = 0.0577). Moreover, in a recent meta-analysis [74] HOMA-IR decreased by 0.51 points per 10 nmol/L increase in endpoint 25OHD among obese patients (*p* = 0.04); the insulin resistance began to decrease at mean level of 25OHD > 70 nmol/L. 

Other studies focused on mainly normal weight healthy children and adolescents [75,76,77,78]. All studies succeeded in significantly increase serum 25OHD levels. Smith et al. [76] supplemented vitamin D_3_ at 10 and 20 µg/day or placebo for 20 weeks in white healthy adolescents in winter at northern latitudes. No differences on cardiovascular risk markers in the fully adjusted analyses for sex, age, Tanner stage, baseline serum 25OHD, and BMI z-score were found. Hauger et al. [75] performed the same RCT in children instead of adolescents. The study showed a marginally significant increase of plasma triglycerides, by 0.03 mmol/L per 10 nmol/L increase in serum 25OHD (*p* = 0.07). This result is in contrast with the supposed beneficial role of vitamin D supplementation on lipid profile and needs to be investigated furtherly. 

Ferira et al. [77] have reported an inverse relationship between vitamin D levels and glucose, insulin, HOMA-IR at baseline. However, they did not find significant difference in these metabolic parameters between groups after supplementation.

Only one study in the past five years showed a significant beneficial effect of vitamin D supplementation on cardiometabolic health in children and adolescents. Tavakoli et al. [78] performed a clinical trial including 47 healthy subjects aged 10–14 years who received vitamin D supplementation (1000 IU/die for one month) or placebo tablets. In the treated group, vitamin D levels increased (*p* = 0.007) as well as serum levels of c-HDL (*p* < 0.001). However, this study has several limitations. The sample was small (only 40 patients reached the follow up), levels of c-HDL were the only cardiometabolic risk factor analyzed, and the duration of intervention was short (4 weeks). 

Table 2 shows the main significant supplementation studies related to vitamin D and cardiovascular risk factors in pediatric age.

None of the studies in obese and normal weight children and adolescents showed symptomatic hypercalcemia or major adverse effects. Nevertheless, a recent meta-analysis [74] found an increase of c-LDL by 0.11 mmol/L with no influence of BMI and baseline serum 25OHD (*p* = 0.002). Another RCT, previously discussed, also observed a possible trend of worsening in lipid profile following vitamin D supplementation [75]. This raises concerns about safety of vitamin D supplementation.

Overall, the results of RCTs do not support vitamin D supplementation for reducing cardiovascular risk in children and adolescents, in accordance with studies conducted in adults. 

## 5. Conclusions

Vitamin D alters the inflammatory response thought different pathways. An inverse correlation between plasma 25OHD levels and cardiovascular risk factors, in particular blood pressure and lipid profile has been evaluated in different studies, also in pediatric age.

However, RCTs studies did not show clear cardiovascular improvements following vitamin D supplementation. A trend of improvement on CV markers was found in two RCTs in obese adolescents with baseline mean vitamin D deficiency. Moreover, a recent meta-analysis show how insulin resistance decreased in overweight/obese children and adolescent after vitamin D supplementation [74].

Limitations of the studies are different: 25OHD baseline levels, duration of intervention, type of vitamin D supplemented (ergocalciferol, cholecalciferol or calcitriol), and quantity administered. In addition, supplementation was administered differently (daily [78,81,82], weekly [83], monthly [84], quarterly [80], or on a single time high dose [79]).

Therefore, further standardized supplementation studies are needed to assess a clear benefit due to vitamin D supplementation in children and adolescents at risk of cardiovascular diseases, such as obese subjects.

## Figures and Tables

**Table 1:** Reviewed studies on Vitamin D deficiency and cardiovascular risk factors in pediatric age. cIMT: carotid intima-media thickness; BMI-z: BMI-for-age z-score; RAR: Retinoic Acid Receptor; RXR: Retinoid X Receptors; aIMT: aortic intima media thickness.

Study	Participants	Vitamin D Deficiency Cut-Off	25OHD Mean Levels	Endpoint	Results
Colak R et al. 2020 [49]	40 obese children (7–14 years) 30 controls (7–14 years)	Deficiency: ≤ 20 ng/mL 25OHD levels	Obese children 16.4 ng/mL Lean children 19.6 ng/mL	Vitamin D levels and lipid profile, fasting glucose and blood pressure Vitamin D levels and ultrasound imaging (cIMT and left ventricular wall thickness)	25OHD concentrations were negatively correlated with 24-h ambulatory blood pressure and cIMT (*p* <0.05) No associations between 25OHD values and fasting plasma glucose, HOMA-IR or lipid profile were present
Censani M et al. 2018 [50]	178 overweight and obese children (6–17 years)	Deficiency: ≤ 20 ng/mL 25OHD levels	20.7 ± 9.2 ng/mL	Vitamin D levels and lipid profile	Patients with 25OHD < 20 ng/mL had significantly higher non-HDL cholesterol, TC, TG and LDL levels (*p* ≤ 0.03) and a significantly higher TG/HDL and TC/HDL ratios (*p* ≤ 0.03)
Iqbal AM et al. 2017 [51]	376 obese children (2–18 years)	Deficiency: ≤ 20 ng/mL 25OHD levels Sufficiency: > 30 ng/mL 25OHD levels	25.2 ± 10.10 ng/mL	Vitamin D levels and lipid profile	25OHD values were negatively associated with BMI z-score (*p* = 0.004) and were positively correlated with c-HDL also after adjustment for age, sex, BMI metric and season of blood draw
Lee M et al. 2016 [52]	209 overweight or obese children (6–18 years)	Deficiency: ≤ 20 ng/mL 25OHD levels	20.3 ± 6.4 ng/mL	Vitamin D levels and lipid profile	A 10 mg/dl increase in TC and oxidated-LDL was associated respectively with a 1,3% and 0,8% decrease in 25OHD concentrations
Petersen R et al. 2015 [55]	782 children (8–11 years)	Deficiency: ≤ 25 nmol/L 25OHD levels	60.8 ± 18.7 nmol/L	Vitamin D levels and glucose concentration, lipid profile, insulin, blood pressure and heart rate weighted for fat mass index	Serum 25OHD was negatively associated with diastolic blood pressure, total and c-LDL, TG and lower metabolic syndrome score, also after adjustment for fat mass index
Kim MR et al. 2019 [56]	243 non-obese healthy volunteers (9–18 years)	Deficiency: ≤ 20 ng/mL 25OHD levels	17.27 ± 6.89 ng/mL	Vitamin D levels and lipid profile	Vitamin D levels significantly inversely associated with TG level and TG/c-HDL ratio (*p* < 0.001)
Liang X et al. 2018 [57]	164 children (6–12 years) Hypertensive vs control subjects	Deficiency: <50 ng/mL 25OHD levels	38.22 ± 12 nmol/L in hypertension group 43.28 ± 12.3 nmol/L in control group	Vitamin D levels and blood pressure, the transcription level of RARs and RXRs, 25OHD receptor	Serum 25OHD in children with hypertension was lower than that in the control group (*p* = 0.02). Serum 25OHD and 25OHD receptor were significantly associated with blood pressure level, and both breastfed and c-HDL were independent protective factors of blood pressure level
Arman D et al. 2019 [59]	135 term healthy neonates	Deficiency: ≤ 20 ng/mL 25OHD levels Sufficiency: > 30 ng/mL 25OHD levels	15.17 ± 9.66 ng/mL	Vitamin D levels and aIMT and cIMT	Neonates with vitamin D sufficiency had a lower aIMT than the others (*p* = 0.001)
Sauder KA et al. 2019 [60]	1410 birth cohort of ethnically diverse pregnant woman and their offspring 715 children evaluated at 4 to 6 years old		Childhood: 55.8± 21.1 nmol/L	Vitamin D levels and CV risk factors (blood pressure, arterial stiffness, body size, and adiposity)	Higher vitamin D levels in cord blood are associated with lower systolic and diastolic blood pressure at 4 to 6 years of age, regardless of childhood 25OHD levels, race/ethnicity, and other covariates
Wang G et al. 2019 [63]	Birth cohort study of 775 children, followed prospectively up to 18 years	Deficiency: < 11 ng/mL on cord blood and < 25 ng/mL in early childhood 25OHD levels	Birth: 13.5 ± 9.9 ng/mL Childhood: 32.6 ± 10.8 ng/mL	Vitamin D levels and blood pressure	Low vitamin D status at birth was associated with higher risk of elevated SBP at ages 3 to 18 years. Low vitamin D status in early childhood was associated with a 1,59-fold higher risk of elevated SBP at age 6 to 18 years
Miliku K et al. 2018 [64]	4903 mother-children pairs re-evaluated at 6 years	Deficiency: <50 ng/mL 25OHD levels	Birth: 28.8 ± 9.9 nmol/L; Childhood: 64 ± 10.8 nmol/L	Vitamin D levels and blood pressure, lipid profile, BMI	25OHD concentrations were not associated with cardiovascular risk factors

**Table 2:** Reviewed studies on vitamin D supplementation and cardiovascular risk factors in pediatric age. WBISI: Whole Body Insulin Sensitivity Index; HbA1c: Glycated haemoglobin; hs-CRP: high-sensitivity C-Reactive Protein.

Study	Where and Season	Participants	Baseline Mean 25OHD Levels	Treatment	Control Group	Duration	Endpoints	Results
Hauger et al. (2018) [75]	Denmark Winter	130 Normal weight children 4–8 years White	10µg/d: 56.9 ± 12.7 nmol/L 20µg/d: 58.1 ± 13.5 nmol/L Control group: 55.2 ± 10.8 nmol/L	D_3_ 10 or 20 µg/day	Placebo	20 weeks	25OHD levels BMI, SBP, DBP, lipid profile, glucose, insulin, HbA1c	25OHD increased to 61.8 ± 10.6 nmol/L in the 10 µg/d group, to 75.8 ± 11.5 nmol/L in the 20 µg/d group No effect on any of the cardiometabolic risk markers Marginal dose-response effect on triglycerides, which increased by 0.03 nmol/L per 10 nmol/L increase in 25OHD (*p* = 0.07)
Smith et al. (2018) [76]	UK Winter	110 Normal weight adolescents 14–18 years White	10µg/d: 49.2 ± 12.0 nmol/L 20µg/d: 51.7 ± 13.4 nmol/L Control group: 46.8 ± 11.4 nmol/L	D_3_ 10 or 20 µg/day	Placebo	20 weeks	25OHD levels BMI, waist circumference, SBP, DBP, glucose, lipid profile	Baseline serum 25OHD was inversely associated with BMIz (*p* < 0.001) and waist circumference (*p* = 0.002) 25OHD increased to 56.6 ± 12.4nmol/L in the 10µg/d group, to 63.9 ± 10.6nmol/L in the 20µg/d group No significant differences in cardiovascular risk factors within either group or between groups
Ferira et al. (2016) [77]	USA Winter	323 Normal weight children and adolescents 9–13 years Mixed	Mean: 70.0 ± 1.0 nmol/L	D_3_ 400,1000, 2000 or 4000 IU/day	Placebo	12 weeks	Dose-response effects of vitamin D on fasting glucose, insulin and HOMA-IR	Baseline 25OHD was inversely associated with BMI (*p* = 0.003), insulin (*p* = 0.005) and HOMA-IR (*p* = 0.012) No significant difference in fasting glucose, insulin and HOMA-IR between groups over time after supplementation
Tavakoli et al. (2016) [78]	Iran Not known	47 Normal weight children and adolescents 10–14 years Caucasian (Iranian)	Treatment group: 7.55 ± 4.96 ng/mL Control group: 9.71 ± 5.48 ng/mL	D 1000IU/day	Placebo	4 weeks	25OHD levels c-HDL	25OHD increased in the treatment group (11.50 ± 5.84ng/mL, *p* < 0.001) c-HDL significantly increased in the treatment group (+ 4.10 ± 6.10mg/dL, *p* = 0.007)
Brar et al. (2018) [79]	Not known All year	20 Obese adolescents 12–18 years Mixed, 75% Hispanic	Mean levels: 16.7 ± 2.9 ng/mL	D_2_ 300000 IU once	Placebo (crossover at week 6)	6 weeks	25OHD levels Insulin metabolism	25OHD treatment group: 19.5 ± 4.5 ng/mL (*p* = 0.0029), control group: 17.2 ± 4.7 ng/mL (p 0.5262) WBISI showed a trend towards improvement in the treated group (*p* = 0.0577)
Shah et al. (2015) [80]	USA All year	40 obese adolescents 11–17 years Mixed	Treatment group: 19.6± 1.4 ng/mL Control group: 25.8 ± 2.6 ng/mL	D_2_ 150000IU baseline and at 12 weeks	Placebo	24 weeks	25OHD levels BMI, lipid profile, HbA1c	Baseline 25OHD was inversely associated with BMI No significant difference in 25OHD levels and no significant changes in any of the markers analysed after vitamin D2 supplementation
Javed et al. (2015) [81]	USA All year	51 Obese adolescents 12–18 years Caucasian	Treatment group: 23.5 ± 8.5 ng/mL Control group: 24.4 ± 7.7 ng/mL	D_3_ 2000 IU/day	400 IU/day	12 weeks	25OHD levels Insulin metabolism Lipid profile	A significant increase in 25OHD in the 2000IU/d group (*p* = 0.04) No change in parameters of insulin metabolism or lipid profile
Magge et al. (2018) [82]	USA All year	26 Obese adolescents 12–17 years African American	Treatment group: 12.3 ± 3.5 ng/mL Control group: 11.7 ± 4.1 ng/mL	D_3_ 5000 IU/day	1000 IU/day	12 weeks	25OHD levels BMI-z, HOMA-IR, lipid profile, hs-CRP	25OHD treatment group: 28.8 ± 11.4 ng/mL (p < 0.0001), control group: 18.8 ± 3.9 ng/mL (*p* = 0.0006) No significant difference in cardiometabolic markers within either group or between groups following Vitamin D3 supplementation
Sethuruman et al. (2018) [83]	USA All year	29 Obese adolescents 13–17 years African American	Treatment group: 12.1 ± 3.8 ng/mL Control group: 12.4 ± 3.8 ng/mL	D_2_ 50000IU once per week + 500 mg/day calcium carbonate	Placebo once per week + 500mg/day calcium carbonate	12 weeks	25OHD levels Insulin metabolism Lipid profile	25OHD treatment group: 32 ng/mL (*p* < 0.0001), control group: 13 ng/mL (*p* = 0.126) 25OHD was positively correlated with HDL (*r* = 0.6, *p* < 0.05) and fasting insulin (*r* = 0.5, *p* < 0.05), but not HOMA-IR (*r* = 0.5, *p* = 0.08)
Varshney et al. (2019) [84]	India All year	202 Obese children and adolescents 11–17 years Asian Indian	Treatment group: 8.36 ± 5.45 ng/mL Control group: 9.01 ± 5.59 ng/mL	D 120,000 IU once a month	12,000 IU once a month	12 months	25OHD levels Insulin metabolism Lipid profile, pulse wave velocity and augmentation index	25OHD treatment group: 26.89 ± 12.23 ng/mL; control group 13.14 ± 4.67 ng/mL (p < 0.001) No changes in insulin metabolism or in cardiovascular risk factors within either group or between groups after supplementation

## Abbreviations

1,25(OH)2D	1,25-dihydroxyvitamin, calcitriol
15-PGDH	15-Hydroxyprostaglandin Dehydrogenase
25OHD	Vitamin D, 25-hydroxyvitamin D
aIMT	Aortic intima media thickness
BMI	Body Mass Index
BMI-z	BMI-for-age z-score
CAD	Coronary artery disease
c-HDL	Cholesterol High Density Lipoprotein
cIMT	Carotid intima-media thickness
c-LDL	Cholesterol Low Density Lipoprotein
COX-2	Cyclooxygenase-2
CV	Cardiovascular
CVD	Cardiovascular disease
CYP27B1	Cytochrome P450 27B1, 1-alpha hydroxylase
DBP	Diastolic blood pressure
HbA1c	Glycated hemoglobin
HDL	High Density Lipoprotein
HOMA-IR	Homeostasis Model Assessment of Insulin Resistance
hs-CRP	high-sensitivity C-Reactive Protein
IMT	Intima-media thickness
LDL	Low Density Lipoprotein
LVH	Left ventricular hypertrophy
PTH	Parathormone
RAAS	Renin-angiotensin-aldosterone system
RAR	Retinoic Acid Receptor
RCT	Randomized controlled trials
RXR	Retinoid X Receptors
SBP	Systolic blood pression
TC	Total cholesterol
TG	Triglycerides
UV	Ultraviolet
VDBP	Vitamin D binding protein
VDR	Vitamin D receptor
VDRE	Vitamin D response element
VEGF	Vascular endothelial growth factor
VSMC	Vascular smooth muscle cell
WBISI	Whole Body Insulin Sensitivity Index
